# Measuring Active Power as the Difference between the Peak Value of Instantaneous Power and the Apparent Power

**DOI:** 10.3390/s22093517

**Published:** 2022-05-05

**Authors:** Giovanni Nobile, Mario Cacciato, Ester Vasta

**Affiliations:** 1 Independent Researcher, 97100 Ragusa, Italy; 2 Department of Electrical, Electronics Engineering and Computer Science (DIEEI), University of Catania, 95100 Catania, Italy; mario.cacciato@unict.it (M.C.); ester.vasta@phd.unict.it (E.V.)

**Keywords:** electric power measurement, active power, instantaneous power, power analyzers

## Abstract

The traditional approach to calculate the active and reactive power in AC power systems requires the measurement of the phase shift between the voltage and current for the evaluation of the power factor. To do this, power analyzers can implement several methods. In principle, it is always necessary to identify specific points of waveforms (e.g., using a zero-crossing detection technique) and get their time shift. In a similar way, the frequency value must be evaluated in order to calculate the angular frequency. Unfortunately, this kind of common method exhibits some issues, such as the large sensitivity to noise. Moreover, inaccuracies in the evaluation of the power factor have a big impact on the final estimation of the electric power. This paper presents a simple but effective way to calculate the electric power, overcoming the need for a direct measurement of the phase shift and frequency. In particular, it is shown that the active power can be easily calculated as the difference between the peak value of the instantaneous power and apparent power. The reactive power and power factor are evaluated by exploiting the same quantities. The practical implementation of the proposed formulation in power analyzers guarantees several benefits without reducing accuracy.

## 1. Background

Power metering is crucial in modern electric systems. The optimal management of AC grids requires the accurate estimation of active power. This is especially true in the case of bidirectional power flows for smart grids where renewable power plants, loads, and storage systems are present at the same time [1,2,3]. Power metering also plays a key role in many other fields, such as power converters, electrical machines, drive systems, electromagnetic compatibility, energy saving, etc. [4,5,6,7].

A wide number of devices designed for measuring active power are available on the market. Some reviews about mainstream measurement technologies are in [1,4,8].

In [8], the authors present a detailed survey on the existing types of power and energy measurement devices while also considering traditional technologies such as electrodynamo wattmeters, thermal wattmeters, induction-type energy meters, and so on. The working principles of these devices and their typical applications are discussed in order to highlight their merits and drawbacks.

Nowadays, electronic power analyzers integrate many functions, providing the measurement of active and reactive power, power factor, THD, etc. They are suitable for steady-state, as well as for transient, conditions. These systems acquire simultaneous samples of voltage and current waveforms, digitize these values, and carry out arithmetic multiplication and averaging using digital techniques to obtain the power measurement [4]. The high-speed acquisition of voltage and current using modern analog-to-digital converters, along with powerful elaboration devices, makes digital analyzers the dominant technology for power metering. This work refers to such systems.

To evaluate the active power, a standard electronic wattmeter calculates both the rms value of voltage and current and the power factor. Focusing on the latter, the traditional approach consists of the evaluation of the phase shift between the voltage and current waveforms. This implies the necessity to directly measure the time shift between the voltage and current waveforms. In principle, it is necessary to detect specific points of waveforms (e.g., using a trigger or a zero-crossing detection technique) and get the period between such points [9,10]. It is also required knowledge of the actual value of the frequency, especially if some variations were expected to take place. The presence of noise or distorted waves could imply a significant error in final estimation for all the applications requiring a precise synchronization [9,11,12].

With the purpose to overcome the need for a direct measurement of phase shift and frequency, this paper presents an alternative way for the evaluation of active power. In particular, this work proves that active power can be expressed as the difference between the peak value of instantaneous power and the apparent power. The reactive power and power factor are calculated by exploiting the same quantities.

In comparison to the traditional approach, the new one is simpler in terms of practical implementation, especially when the voltage and current are pure sine waves. Moreover, in most cases, accuracy is very high even in the presence of noise or distorted waves.

Section 2 reports the main relationships representing the core of this work. Section 3 provides the analytical proof of the new formulation. In Section 4, validation is discussed, with additional information about measurement errors, three-phase systems, and harmonic disturbances. Section 5 lists the main advantages of the proposed method. 

All the results reported in this paper do not take into account the presence of harmonics in voltage and current waveforms, except for Section 4.3, which provides some brief information about harmonic distortion. A comprehensive analysis about the calculation of electric power in the case of harmonics is out of the scope of this work.

Although this study deals with AC electric systems, it is worth noting that there are other possible fields of application, for example, mechanical vibrations, complex analysis, and so on.

## 2. Introducing the New Method for Measuring Electric Power

For the sake of simplicity and clarity, this section refers to a single-phase electric circuit in a steady-state condition.

The core of this paper consists of the following three equations:

(1) $$\cos\varphi =\frac{p_{M}}{S}-1$$

(2) $$P=p_{M}-S$$

(3) $$\left|Q\right|=\sqrt{p_{M}\cdot \left(2\cdot S-p_{M}\right)}$$

where *φ* is the phase difference between the voltage and current, *cosφ* is the power factor, *P* is the active power, *Q* is the reactive power, *S* is the apparent power and *p_M_* is the peak value of the instantaneous power.

The main result is the calculation of the active power *P* in Equation (2) as the difference between the peak value of the instantaneous power *p_M_* and the value of the apparent power *S*. In this way, no physical measurement of frequency or phase shift is required because the evaluation of the power factor becomes unnecessary for the determination of the active power. At the same time, the calculation of the power factor and reactive power in the new approach comes from the peak value of the instantaneous power.

We note that in Equation (3), the reactive power *Q* is an absolute value since it is expressed as a square root. Alternatively, for the range *φ* ∈ [0, π], one can calculate *φ* from the power factor in Equation (1) and then calculate *Q* using *sinφ*. 

**Figure 1:**
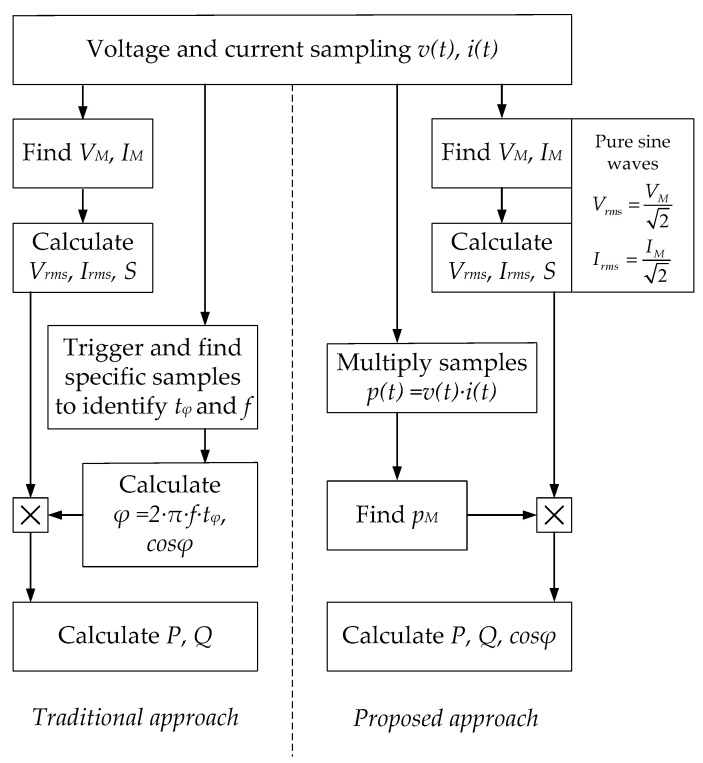
Traditional vs. new approach.

From a practical point of view, in comparison to the traditional measurement process, the proposed formulation provides a simpler way to get the electric power, as highlighted by the flow chart in Figure 1.

The main advantage is the opportunity to avoid the direct measurement of the time interval *t_φ_* and of the frequency *f*, which are required in the traditional formulation to evaluate *cosφ*, as follows:

(4) $$\varphi =w\cdot t_{\varphi }=2\cdot \pi \cdot f\cdot t_{\varphi }\Rightarrow \cos\varphi$$

where *ω* is the angular frequency.

This means that common algorithms used by power analyzers, scopes, etc. for measuring phase shift could be no longer required, especially when the final target is the evaluation of the active or reactive power.

At the same time, the issues linked to traditional algorithms become irrelevant. An example is the uncertainty in zero-crossing detection in the case of noise superimposed to the voltage or current waveform. This uncertainty can cause a significant error in the evaluation of the phase shift and, consequently, in the final value of the active power. On the contrary, the new method does not require any zero-crossing.

Measurement of voltage and current peak values can be carried out using one of the common methods in the literature. The calculation of rms values is immediate in the case of pure sine waves, and so it is easy to get the value of *S*.

More details about the advantages of the proposed approach are discussed in Section 5.

## 3. Analytical Proof

Considering a standard single-phase electric circuit in a steady-state condition where the voltage and the current are pure sine waves, the phase difference between such signals is the angle *φ*:

(5) 0≤φ<2π

The basic equations in the time domain are:

(6) $$\begin{array}{l} v(t)=V_{M}\cdot \cos(\omega \cdot t)\\ i(t)=I_{M}\cdot \cos(\omega \cdot t+\varphi ) \end{array}$$

where *V_M_* and *I_M_* are the peak values of voltage and current, respectively.

By definition, the instantaneous power is:

(7) p(t)=v(t)·i(t)

thus:

(8) $$p(t)=V_{M}\cdot I_{M}\cdot \cos(\omega \cdot t)\cdot \cos(\omega \cdot t+\varphi )$$

**Figure 2:**
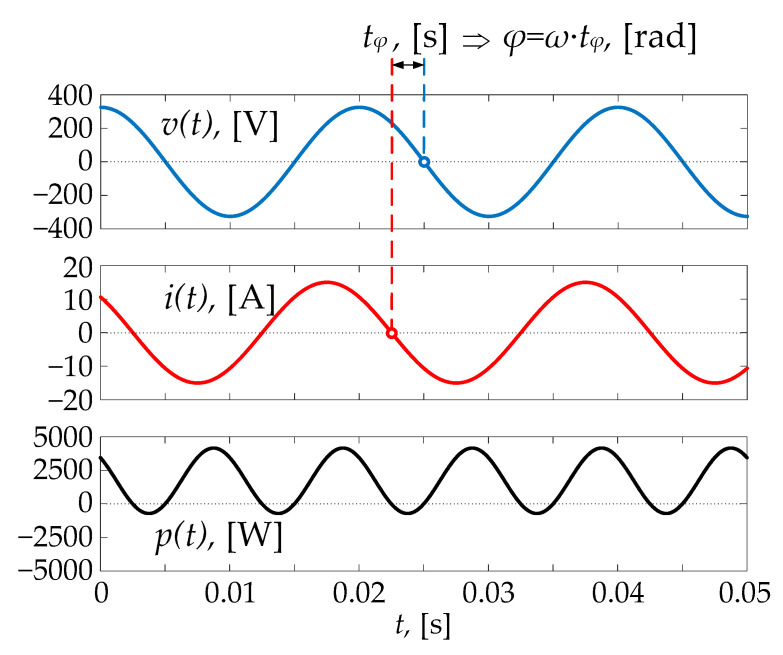
Example of waveforms where the frequency is 50 Hz, time delay is 0.0025 s, and phase shift is 0.25π rad.

For the sake of example, Figure 2 shows voltage, current, and instantaneous power waveforms.

Using Werner formulas, Equation (8) becomes:

(9) $$\begin{array}{l} p(t)=V_{M}\cdot I_{M}\cdot \frac{1}{2}\cdot \left[\cos(2\cdot \omega \cdot t+\varphi )+\cos(-\varphi )\right]\\ =\frac{1}{2}\cdot V_{M}\cdot I_{M}\cdot \left[\cos(2\cdot \omega \cdot t+\varphi )+\cos(\varphi )\right] \end{array}$$

since:

(10) cos(−φ)=cos(φ)

Focusing on the peak value of the instantaneous power and searching for its analytical expression, it is necessary to calculate the time derivative of *p*(*t*) from Equation (9) and set such derivative equal to zero:

(11) $$\frac{dp(t)}{dt}=\frac{1}{2}\cdot V_{M}\cdot I_{M}\cdot \left[-\sin(2\cdot \omega \cdot t+\varphi )\cdot 2\cdot \omega \right]=0$$

**Figure 3:**
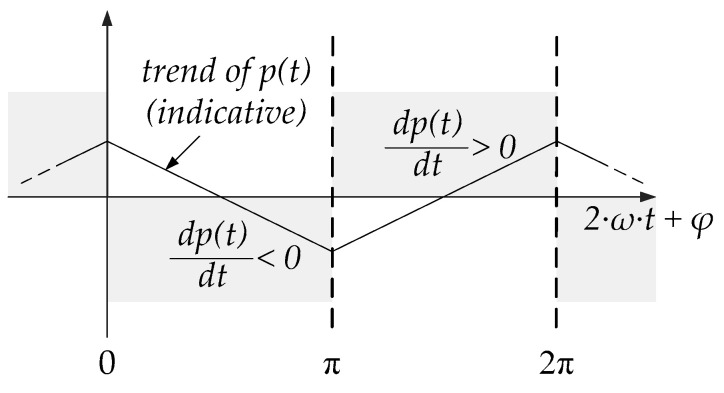
Trend of instantaneous power and its time derivative in Equations (9) and (11).

To identify the maximum points while excluding the minimum ones, the graph of *p*(*t*) and its time derivative can be useful, as seen in Figure 3. The argument of sine in Equation (11) is in the horizontal axis.

The time derivative of the instantaneous power in (11) is positive when:

(12) −sin(2·ω·t+φ)>0

or:

(13) sin(2·ω·t+φ)<0

This is true in the range:

(14) π<2·ω·t+φ<2π

Looking at Figure 3, the maximum point for the instantaneous power occurs at 2π or 0, and thus when the argument of the sine is:

(15) 2·ω·t+φ=2π=0

Considering this point, a further confirmation comes from the calculation of the second derivative of the instantaneous power:

(16) $$\frac{d^{2}p(t)}{dt^{2}}=-\omega \cdot V_{M}\cdot I_{M}\cdot \cos\left(2\cdot \omega \cdot t+\varphi \right)\cdot 2\cdot \omega$$

that is negative:

(17) $$\frac{d^{2}p(t)}{dt^{2}}=-\omega \cdot V_{M}\cdot I_{M}\cdot 2\cdot \omega <0$$

because, from Equation (15):

(18) cos(2·ω·t+φ)=cos(2π)=cos(0)=1

The second derivative is negative, confirming the detection of the maximum point for the instantaneous power.

Coming back to Equation (9), the instantaneous power at the maximum point is expressed as:

(19) $$p(t)=\frac{1}{2}\cdot V_{M}\cdot I_{M}\cdot \left[\cos(2\cdot \omega \cdot t+\varphi )+\cos(\varphi )\right]=\frac{1}{2}\cdot V_{M}\cdot I_{M}\cdot \left[1+\cos(\varphi )\right]$$

or:

(20) $$p_{M}=\frac{1}{2}\cdot V_{M}\cdot I_{M}\cdot \left[1+\cos(\varphi )\right]$$

then the power factor *cosφ* is:

(21) $$\cos\varphi =\frac{p_{M}}{V_{M}\cdot I_{M}}\cdot 2-1$$

Peak values of voltage and current can be rewritten in terms of rms values, as follows:

(22) $$V_{M}=\sqrt{2}\cdot V_{rms}\;I_{M}=\sqrt{2}\cdot I_{rms}$$

so that:

(23) $$\cos\varphi =\frac{p_{M}}{V_{rms}\cdot I_{rms}}-1$$

or:

(24) $$\cos\varphi =\frac{p_{M}}{S}-1$$

From a mathematical point of view, these relations are undefined in the case of zero value for *V_rms_* or *I_rms_*. Regardless, in this case, the electric power is null for certain, and no further calculations are necessary.

Finally, multiplying each term of Equation (24) by *S* and considering that the active power *P* is the product of *S* and *cosφ*, the new formulation for the evaluation of the active power is obtained by:

(25) $$P=p_{M}-S$$

Thus, the active power can be easily calculated as the difference between the peak values of the instantaneous power and apparent power. The last equation suggests that the active power can be computed without measuring the phase shift and frequency.

From power triangle formula:

(26) $$S^{2}=P^{2}+Q^{2}$$

the absolute value of the reactive power is easily obtained by the following:

(27) $$\left|Q\right|=\sqrt{p_{M}\cdot \left(2\cdot S-p_{M}\right)}$$

Alternatively, in the range *φ* ∈ [0, π], one can calculate *φ* from the power factor in Equation (24) and then calculate *Q* using *sinφ*.

The final results remain the same in the case of voltage or current expressed in terms of sine, as in Equation (6). An analytical proof is in Appendix A.

## 4. Validation

It is easy to verify that the proposed formulas used to calculate the active and reactive power lead to the same values achieved by using traditional equations.

To validate this statement, readers can implement a simple code in Matlab environment as the one reported in the following, confirming the correctness of the proposed formulation. This example refers to a pure sine waves condition.

*%%%%%%%%%%%%%%%%%%%%%%%%%%%%%%%%%%%%%%%%%*

*t = 0:1e-6:0.05; % time samples*

*f = 50; % frequency*

w = 2*pi*f; % angular frequency

phi = 1/4*pi; % phase shift between voltage and current

*VM = 325; % voltage peak value*

*IM = 15; % current peak value*

*Vrms = VM/sqrt(2); % voltage rms value*

*Irms = IM/sqrt(2); % current rms value*

v = VM*cos(w*t); % instantaneous voltage

i = IM*cos(w*t + phi); % instantaneous current

p = v.*i; % instantaneous power

*pM=max(p); % power peak value*

*plot(t,v,t,i,t,p); % plot waveforms*

S = Vrms*Irms; % apparent power

*cosphi_standard=cos(phi) % power factor*

P_trad = S*cos(phi) % active power, traditional calculation

Q_trad = S*sin(phi) % absolute value of reactive power, traditional calculation

*cosphi_new = pM/S-1 % power factor calculation, new approach*

*P_new = pM-S % active power calculation, new approach*

Q_new = sqrt(pM*(2*S-pM)) % absolute value of reactive power calculation, new approach

*%%%%%%%%%%%%%%%%%%%%%%%%%%%%%%%%%%%%%%%%%*

The following sections report information about:

- An analysis of measurement errors in the case of noise;
- The implementation of the proposed method for three-phase systems; and
- The implementation in the case of harmonic distortion.

The theoretical analysis for each of these cases can be easily confirmed by simulating different scenarios and comparing the results coming from the new method to the ones obtained with the standard approach.

In recent years, some experimental tests in different test benches have been carried out. To this aim, specific prototypal boards for the sensing and conditioning of signals have been developed to realize three-phase digital power meters for specific applications [13,14]. Control has been realized using different types of micro-controllers. In all scenarios, at different voltage amplitude, frequency, noise, harmonics, etc., the results confirm the validity of the theoretical analysis.

From a practical point of view, during laboratory activities, it has been observed that the proposed method can be easily implemented by programming commercial, low-cost micro-controllers. One of the main advantages consists in avoiding the use of any counter subroutine for the evaluation of the frequency or phase shift.

### 4.1. Error Analysis

In many operating conditions, the relative error of the proposed method is expected to be lower in comparison to the traditional approach for the following reasons.

The first reason deals with the number of elements whose relative error impacts the overall inaccuracy. Active power is commonly expressed as:

(28) $$P=V_{rms}\cdot I_{rms}\cdot \cos\varphi =V_{rms}\cdot I_{rms}\cdot \cos\left(\omega \cdot t_{\varphi }\right)=V_{rms}\cdot I_{rms}\cdot \cos\left(2\cdot \pi \cdot f\cdot t_{\varphi }\right)$$

Based on the rules for the propagation of uncertainties, the relative error *ε_P_* linked to the final value of *P* is proportional to the relative error caused by each element:

(29) $$\varepsilon_{P}\propto \left(\varepsilon_{V_{rms}},\varepsilon_{I_{rms}},\varepsilon_{f},\varepsilon_{t_{\varphi }}\right)$$

where each term is the ratio between the absolute error Δ and the value of the physical quantity:

(30) $$\varepsilon_{V_{rms}}=\frac{\Delta_{V_{rms}}}{V_{rms}}\;\;\varepsilon_{I_{rms}}=\frac{\Delta_{I_{rms}}}{I_{rms}}\;\;\varepsilon_{f}=\frac{\Delta_{f}}{f}\;\;\varepsilon_{t_{\varphi }}=\frac{\Delta_{t_{\varphi }}}{t_{\varphi }}$$

In the new approach, active power is calculated as in Equation (2):

(31) $$P=p_{M}-S=p_{M}-V_{rms}\cdot I_{rms}$$

so that:

(32) $$\varepsilon_{P}\propto \left(\varepsilon_{p_{M}},\varepsilon_{V_{rms}},\varepsilon_{I_{rms}}\right)$$

where:

(33) $$\varepsilon_{p_{M}}=\frac{\Delta_{p_{M}}}{p_{M}}$$

In comparison to Equation (29), which is referred to the traditional formula, the number of relative error terms in Equation (32) is lower. In other words, the number of elements that would lead to inaccuracies are reduced.

In most real applications, the power factor is close to 1, or it is compensated in order to get high values for well-known reasons [15,16]. This means that the phase difference between the voltage and current is usually close to 0 or very low. In this case, the value of *p_M_* in Equation (33) is very high, leading to the opportunity to neglect its relative error in Equation (32).

**Figure 4:**
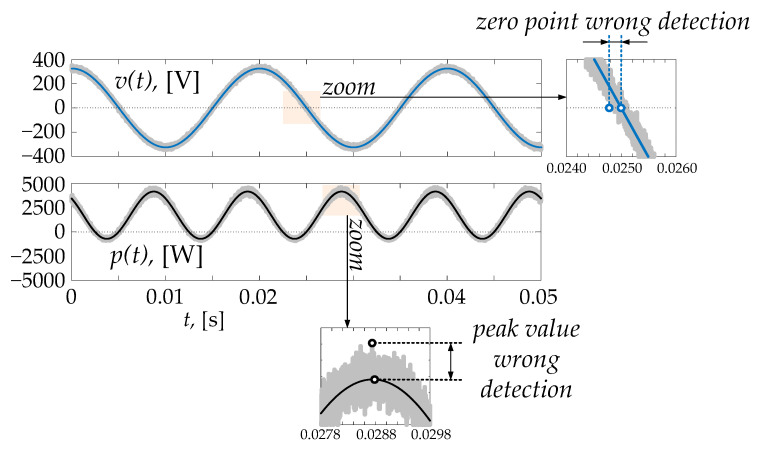
Measurement issues in the case of white Gaussian noise, where the signal-to-noise ratio is 30.

A rigorous analysis of the measurement errors taking into account all possible scenarios, as well as the systematic and accidental errors, is out of the scope of this work. Regardless, from the qualitative analysis reported above, it is clear that the proposed method gives some benefits for accuracy. For the sake of example, Figure 4 shows voltage and instantaneous power in the presence of white Gaussian noise [17], where the signal-to-noise ratio is 30. Zoom windows highlight the issues encountered during the measurement process, assuming that the instrument adopts the first sample that reaches the zero value as the instant of the zero-cross of the waveform. In this numerical example, the rated values are:

(34) $$V_{M}=325.00\;V,\;V_{rms}=229.81\;V\;I_{M}=15.00\;A,\;I_{rms}=10.61\;A\;t_{\varphi }=0.00250\;s,\;f=50.00\;\mathrm{Hz},\;\varphi =0.25\;\pi \;\mathrm{rad}=0.7854\;\mathrm{rad}\;P=V_{rms}\cdot I_{rms}\cdot \cos\varphi =1723.6\;W\;$$

Using the traditional formula, in the worst case the measurements, errors caused by noise lead to:

(35) $$V_{M}=348.50\;V,\;V_{rms}=246.43\;V\;I_{M}=15.95\;A,\;I_{rms}=11.28\;A\;t_{\varphi }=0.00258\;s,\;f=50.30\;\mathrm{Hz},\;\varphi =0.8154\mathrm{rad}\;P=V_{rms}\cdot I_{rms}\cdot \cos\varphi =1905.7\;W,\underline{\varepsilon_{P}=0.1057≅11\%}$$

Repeating the measurement with the proposed method and using for the calculation the sample of *p*(*t*) with the highest recorded value, the final relative error in the worst case is lower:

(36) $$V_{M}=348.50\;V,\;V_{rms}=246.43\;V\;I_{M}=15.95\;A,\;I_{rms}=11.28\;A\;p_{M}=4578.3\;W\;P=p_{M}-S=p_{M}-V_{rms}\cdot I_{rms}=1798.5\;W,\underline{\varepsilon_{P}=0.0435≅4\%}$$

This example confirms the effectiveness of the proposed approach for the final accuracy.

### 4.2. Three-Phase Systems 

In a three-phase system, Equation (2) for the active power becomes:

(37) $$P=p_{M,a}-S_{a}+p_{M,b}-S_{b}+p_{M,c}-S_{c}$$

where the terms *p_M_* and *S* are referred to the phases *a*, *b*, and *c*. In a symmetrical and balanced three-phase system, a compact form is obtained as follows:

(38) $$P=3\cdot (p_{M}-S)$$

where *p_M_* and *S* are again referred to a single phase. The power factor and reactive power are:

(39) $$\cos\varphi =\frac{p_{M}}{S}-1$$

(40) $$\left|Q\right|=3\cdot \sqrt{p_{M}\cdot \left(2\cdot S-p_{M}\right)}$$

### 4.3. Harmonics

The increasing presence of renewable energy sources, storage systems, and power electronics devices into the grid causes a significant harmonic distortion in the current and voltage waveforms [18,19,20]. Modern power meters shall be able to measure the active power in the presence of harmonics. 

The proposed method also remains valid in this case. Equation (2) has to be applied for each harmonic component *h*:

(41) $$P_{h}=p_{M,h}-S_{h}=p_{M,h}-V_{rms,h}\cdot I_{rms,h}$$

The total power is the sum of the active power contributions from all the *H* harmonics:

(42) $$P=\sum_{H}P_{h}$$

or:

(43) $$P=\sum_{H}p_{M,}{}_{h}-V_{rms,t}\cdot I_{rms,t}$$

where *V_rms,t_* and *I_rms,t_* are the total rms values of voltage and current:

(44) $$V_{rms,t}=\sqrt{\frac{1}{H}\cdot \sum_{H}V_{rms,h}^{2}}\;\;\;I_{rms,t}=\sqrt{\frac{1}{H}\cdot \sum_{H}I_{rms,h}^{2}}$$

## 5. Discussion

Below is a summary of benefits and other information related to the method presented in this paper:

- Active power is calculated in a straightforward way by exploiting the peak values of the instantaneous power and apparent power;
- The presented approach is similar to the traditional one, but its practical implementation is simpler;
- A direct measurement of the phase shift between signals (i.e., the direct measurement of their time delay) is no longer required;
- A direct measurement of frequency is no longer required;
- In the case of frequency or amplitude variations, the estimation of the active power is automatically updated after a brief settling time;
- In comparison to the traditional procedure, the proposed method ensures the reduction of measurement errors thanks to the reduced number of elements impacting the overall inaccuracy and to the opportunity to neglect the relative error of *p_M_* in most cases, as described in Section 4.1;
- It is easy to apply the same formulation to get the active power in three-phase electric systems, as well as in presence of harmonic distortion;
- Taking into account the measurement of the reactive power at the grid level [18], the contribution of this work is a bit limited because *Q* is not calculated in a direct way since it depends on the preliminary assessment of *P*;
- The proposed approach has general validity, and it can be extended for all cases in which it is relevant to analyze the correlation between two or more periodic signals having a time delay; and
- Although this study deals with electric systems, there are other possible fields of application, for example, mechanical vibrations, complex analysis, and so on.

Future works will regard the extension of this study for the cases listed in the last points, including further investigation on the measurement of reactive power.

## 6. Patents

A utility model patent is pending.

## Figures and Tables

**Figure A1:**
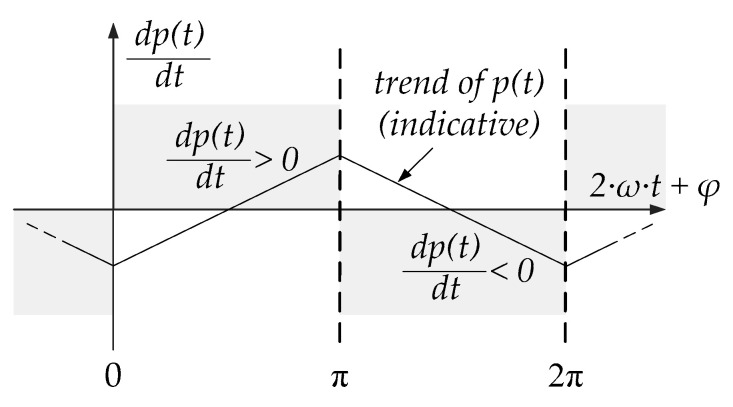
Trend of instantaneous power and its time derivative in Equations (A2) and (A3).

## Data Availability

All data presented in this work are available upon request.

## Appendix A

This appendix reports the equations representing the analytical proof of the proposed method in the case of voltage and current expressed as sine waves. Figure A1 shows the graph of *p*(*t*) and its time derivative. The argument of sine in Equation (A3) is in the horizontal axis.

It is worth noting that the final results are identical to the ones presented in Section 3.

(A1) $$\begin{array}{l} v(t)=V_{M}\cdot \sin(\omega \cdot t)\\ i(t)=I_{M}\cdot \sin(\omega \cdot t+\varphi ) \end{array}$$

(A2) $$\begin{array}{l} p(t)=v(t)\cdot i(t)=V_{M}\cdot \sin(\omega \cdot t)\cdot I_{M}\cdot \sin(\omega \cdot t+\varphi )\\ =\frac{1}{2}\cdot V_{M}\cdot I_{M}\cdot \left[-\cos\left(2\cdot \omega \cdot t+\varphi \right)+\cos\left(-\varphi \right)\right]\\ =\frac{1}{2}\cdot V_{M}\cdot I_{M}\cdot \left[-\cos\left(2\cdot \omega \cdot t+\varphi \right)+\cos\left(\varphi \right)\right] \end{array}$$

(A3) $$\frac{dp(t)}{dt}=\frac{1}{2}\cdot V_{M}\cdot I_{M}\cdot \left[\sin\left(2\cdot \omega \cdot t+\varphi \right)\cdot 2\cdot \omega \right]>0$$

(A4) sin(2·ω·t+φ)>0

(A5) 0<2·ω·t+φ<π

(A6) $$\frac{d^{2}p(t)}{dt^{2}}=\omega \cdot V_{M}\cdot I_{M}\cdot \cos\left(2\cdot \omega \cdot t+\varphi \right)\cdot 2\cdot \omega$$

(A7) $${\frac{d^{2}p(t)}{dt^{2}}|}_{2\cdot \omega \cdot t+\varphi =\pi }=\omega \cdot V_{M}\cdot I_{M}\cdot \cos\left(\pi \right)\cdot 2\cdot \omega <0$$

(A8)
$$\begin{array}{l} {p(t)|}_{2\cdot \omega \cdot t+\varphi =\pi }=p_{M}=\frac{1}{2}\cdot V_{M}\cdot I_{M}\cdot \left[-\cos\left(\pi \right)+\cos\left(\varphi \right)\right]\\ =\frac{1}{2}\cdot V_{M}\cdot I_{M}\cdot \left[1+\cos\left(\varphi \right)\right] \end{array}$$

(A9)
$$\cos\varphi =\frac{p_{M}}{V_{M}\cdot I_{M}}\cdot 2-1=\frac{p_{M}}{S}-1$$

(A10)
$$P=p_{M}-S$$

(A11)
$$\left|Q\right|=\sqrt{p_{M}\cdot \left(2\cdot S-p_{M}\right)}$$

## References

[1] Avancini D.B., Rodrigues J.J.P.C., Martins S.G.B., Rabêlo R.A.L., Al-Muhtadi J., Solic P. (2019). Energy Meters Evolution in Smart Grids: A review. J. Clean. Prod..

[2] Peretto L. (2010). The role of measurements in the smart grid era. IEEE Instr. Meas. Mag..

[3] Nobile G., Vasta E., Cacciato M., Scarcella G., Scelba G., Di Stefano A.G.F., Leotta G., Pugliatti P.M., Bizzarri F. (2020). Performance Assessment of Large Photovoltaic (PV) Plants Using an Integrated State-Space Average Modeling Approach. Energies.

[4] Xiao C., Chen G., Odendaal W.G.H. (2007). Overview of Power Loss Measurement Techniques in Power Electronics Systems. IEEE Trans. Ind. Appl..

[5] De Santis M., Agnelli S., Patanè F., Giannini O., Bella G. (2018). Experimental Study for the Assessment of the Measurement Uncertainty Associated with Electric Powertrain Efficiency Using the Back-to-Back Direct Method. Energies.

[6] Cacciato M., Finocchiaro L., Nobile G., Scarcella G., Scelba G. Assessment of energy management strategies for battery assisted solar pumping systems. Proceedings of the 2016 International Symposium on Power Electronics, Electrical Drives, Automation and Motion (SPEEDAM).

[7] Andò B., Baglio S., Pistorio A., Tina G.M., Ventura C. (2015). Sentinella: Smart Monitoring of Photovoltaic Systems at Panel Level. IEEE Trans. Instrum. Meas..

[8] Babuta A., Gupta B., Kumar A., Ganguli S. (2021). Power and energy measurement devices: A review, comparison, discussion, and the future of research. Measurement.

[9] Mahmud S.M. (1989). Error Analysis of Digital Phase Measurement of Distorted Waves. IEEE Instr. Meas..

[10] Fahim S.R., Avro S.S., Sarker S.K., Das S.K. (2019). A novel fractional order power factor measurement. SN Appl. Sci..

[11] Bartolomei L., Cavaliere D., Mingotti A., Peretto L., Tinarelli R. (2020). Testing of Electrical Energy Meters Subject to Realistic Distorted Voltages and Currents. Energies.

[12] Sanchez A., de Castro A., López-Colino F., Garrido J. Comparison of AC mains synchronization methods when using precalculated duty cycles in power factor correction. Proceedings of the 2014 IEEE 15th Workshop on Control and Modeling for Power Electronics (COMPEL).

[13] Graziani S., Nobile G., Pitrone N., Savalli N. Education enhancement on three-phase system measurements. Proceedings of the 2007 4th WSEAS/IASME International Conference on Engineering.

[14] Nobile G., Scelba G., Cacciato M., Scarcella G. Losses minimization control for an integrated multi-drives topology devoted to hybrid electric vehicles. Proceedings of the 2017 IECON 43rd Annual Conference of the IEEE Industrial Electronics Society.

[15] Cano Ortega A., Sánchez Sutil F.J., De la Casa Hernández J. (2019). Power Factor Compensation Using Teaching Learning Based Optimization and Monitoring System by Cloud Data Logger. Sensors.

[16] Kabir Y., Mohsin M., Khan M.M. Automated power factor correction and energy monitoring system. Proceedings of the 2017 Second International Conference on Electrical, Computer and Communication Technologies (ICECCT).

[17] Xi Y., Li Z., Zeng X., Tang X., Liu Q., Xiao H. (2018). Detection of power quality disturbances using an adaptive process noise covariance Kalman filter. Digital Signal Proc..

[18] Demoulias C.S., Malamaki K.-N.D., Gkavanoudis S., Mauricio J.M., Kryonidis G.C., Oureilidis K.O., Kontis E.O., Martinez Ramos J.L. (2020). Ancillary Services Offered by Distributed Renewable Energy Sources at the Distribution Grid Level: An Attempt at Proper Definition and Quantification. Appl. Sci..

[19] Malamaki K.N., Gkavanoudis S., Mushtaq U., Cvetkovic M., Marano A., Gallos K., Dikaiakos C., Jerele M., Schneider C., Damböck R. (2018). D1.1 Description of the Metrics Developed for the Quasi-Steady-State Operation and Report on the Review of the Respective Current Grid Codes.

[20] Chica Leal A.d.J., Trujillo Rodríguez C.L., Santamaria F. (2020). Comparative of Power Calculation Methods for Single-Phase Systems under Sinusoidal and Non-Sinusoidal Operation. Energies.

